# Comparative Effects of Domain-Specific Human Monoclonal Antibodies Against LGI1 on Neuronal Excitability

**DOI:** 10.1212/NXI.0000000000200096

**Published:** 2023-04-07

**Authors:** Josefine Sell, Vahid Rahmati, Marin Kempfer, Sarosh R. Irani, Andreas Ritzau-Jost, Stefan Hallermann, Christian Geis

**Affiliations:** From the Section Translational Neuroimmunology (J.S., V.R., M.K., C.G.), Department of Neurology, Jena University Hospital, Germany; Oxford Autoimmune Neurology Group (S.R.I.), Nuffield Department of Clinical Neurosciences, University of Oxford, UK; Department of Neurology (S.R.I.), Oxford University Hospitals, UK; and Carl-Ludwig-Institute of Physiology (A.R.-J., S.H.), Faculty of Medicine, Leipzig University, Germany.

## Abstract

**Background and Objectives:**

Autoantibodies to leucine-rich glioma inactivated protein 1 (LGI1) cause an autoimmune limbic encephalitis with frequent focal seizures and anterograde memory dysfunction. LGI1 is a neuronal secreted linker protein with 2 functional domains: the leucine-rich repeat (LRR) and epitempin (EPTP) regions. LGI1 autoantibodies are known to interfere with presynaptic function and neuronal excitability; however, their epitope-specific mechanisms are incompletely understood.

**Methods:**

We used patient-derived monoclonal autoantibodies (mAbs), which target either LRR or EPTP domains of LGI1 to investigate long-term antibody-induced alteration of neuronal function. LRR- and EPTP-specific effects were evaluated by patch-clamp recordings in cultured hippocampal neurons and compared with biophysical neuron modeling. K_v_1.1 channel clustering at the axon initial segment (AIS) was quantified by immunocytochemistry and structured illumination microscopy techniques.

**Results:**

Both EPTP and LRR domain-specific mAbs decreased the latency of first somatic action potential firing. However, only the LRR-specific mAbs increased the number of action potential firing together with enhanced initial instantaneous frequency and promoted spike-frequency adaptation, which were less pronounced after the EPTP mAb. This also led to an effective reduction in the slope of ramp-like depolarization in the subthreshold response, suggesting K_v_1 channel dysfunction. A biophysical model of a hippocampal neuron corroborated experimental results and suggests that an isolated reduction of the conductance of K_v_1-mediated K^+^ currents largely accounts for the antibody-induced alterations in the initial firing phase and spike-frequency adaptation. Furthermore, K_v_1.1 channel density was spatially redistributed from the distal toward the proximal site of AIS under LRR mAb treatment and, to a lesser extant, under EPTP mAb.

**Discussion:**

These findings indicate an epitope-specific pathophysiology of LGI1 autoantibodies. The pronounced neuronal hyperexcitability and SFA together with dropped slope of ramp-like depolarization after LRR-targeted interference suggest disruption of LGI1-dependent clustering of K^+^ channel complexes. Moreover, considering the effective triggering of action potentials at the distal AIS, the altered spatial distribution of K_v_1.1 channel density may contribute to these effects through impairing neuronal control of action potential initiation and synaptic integration.

Autoantibodies against leucine-rich glioma inactivated protein 1 (LGI1 antibody encephalitis) cause limbic encephalitis with frequent focal and generalized acute symptomatic seizures followed by anterograde amnesia.^1-3^ Focal seizures manifest as faciobrachial dystonic seizures, which are nearly pathognomonic for LGI1 antibody encephalitis, alongside several other semiologies.^4-7^ Seizures are usually refractory to antiseizure medications but are typically rapidly responsive to immunotherapies.^8^ Patients harbor antibodies against LGI1 in serum and CSF and may develop bilateral hippocampal atrophy, persistent memory loss, and temporal lobe epilepsy, along with poorer control of acute symptomatic seizures, when the initiation of immunotherapy is delayed.^5,8,9^

LGI1 is a neuronal secreted 60-kDa glycoprotein with a leucine-rich repeat (LRR) and an epitempin (EPTP) domain.^10,11^ Recent imaging data of fluorescently labeled LGI1 argue against secretion but for cycling of LGI1 by exo- and endocytosis.^12^ The homodimerization of LGI1 is mediated by mutual binding of the LRR domain of one LGI1 molecule to the EPTP domain of the second LGI1 and the EPTP propeller structure interacts directly with their receptors: ADAM22 and ADAM23.^13^ Hence, LGI1 is proposed to serve as a transsynaptic linker molecule connecting presynaptic voltage-gated potassium channels of K_v_1.1 type and postsynaptic α-amino-3-hydroxy-5-methyl-4-isoxazolepropionic acid (AMPA) receptors in a multiprotein complex.^11,14,15^ Previous studies found that polyclonal serum LGI1 antibodies from patients with encephalitis and seizures are directed to both the LRR and the EPTP domains and can interfere with LGI1's synaptic linker function, thus reducing the expression of presynaptic K_v_1.1 channels and postsynaptic AMPA receptors.^16,17^ In addition to its role in transsynaptic linkage of excitatory synapses, LGI1 is also important for regulating intrinsic neuronal excitability. LGI1 deletion results in reduction of axonal K_v_1 channels, in particular at the axonal initial segments (AISs) and juxtaparanodes.^18,19^ This parallel reduction of axonal LGI1 and K_v_1 induced an increase of intrinsic excitability, with increased neuronal spiking. Recently, epitope-specific monoclonal antibodies (mAbs) have been derived from the blood and CSF B cells in patients with LGI1 antibody encephalitis.^20,21^ This has shown that EPTP-targeting mAbs inhibited binding of LGI1 to its ADAM22/23 adaptor proteins, whereas LRR-specific antibodies facilitated the internalization of ADAM22/23-bound LGI1.^20,21^ Similarly to LGI1 antibodies purified from patient serum, mAbs targeting both LGI1 subdomains were able to enhance glutamatergic transmission and to increase cellular excitability.^16,20^

Here, we used human mAbs specifically directed against either the EPTP or the LRR domain of LGI1 to directly compare subdomain-specific antibody effects on the intrinsic neuronal excitability and K_v_1.1 channel clustering and spatial distribution at the AIS in dissociated murine hippocampal neurons. We performed long-term incubation of mAbs over 7 days to more closely mimic the effects of LGI1 antibodies in patients where pathogenic autoantibodies likely present for days to weeks before development of characteristic disease symptoms. Moreover, we applied a biophysical neuron model to simulate incremental K_v_1 channel dysfunction and to compare these effects with our experimental findings using subdomain-specific LGI1 mAbs.

## Methods

### Hippocampal Cell Culture Preparation

Primary hippocampal neurons were prepared from E18 embryos of 5 female C57BL/6J mice. Animal breeding and experiments were performed in accordance with the Animal Research: Reporting of In Vivo Experiments (ARRIVE) guidelines for reporting animal research.^22^ Brains were removed from the skull and meninges with fine forceps. Hippocampi were dissected and collected in 37°C Hank balanced salt solution before the tissue was broken down enzymatically in 0.25% trypsin solution for 5 minutes at 37°C and triturated. Dissociated neurons were seeded on poly-d-lysine–coated coverslips in plating medium (0.5% glucose, 10% horse serum, and 0.5% penicillin/streptavidin) with a density of 18.000 cells/cm^2^. One hour after plating, the medium was replaced (with Neurobasal, 2% B27 supplement [Life Technologies], 1% glutamine, 0.5% penicillin/streptavidin), and neurons were cultured for 14–21 days at 37°C before use.

### Monoclonal Antibodies and Cell Treatment

For this study, we used LGI1-specific mAbs, which were described previously.^21^ They are derived from 2 different patients with LGI1-antibody encephalitis and isolated after in vitro activation and differentiation of peripheral blood mononuclear cells to antibody-secreting cells.^21^ Epitope specificity to LRR- and EPTP-domains of LGI1 was characterized using a HEK293T cell–based assay expressing full-length LGI1, LRR, or EPTP domain constructs. An isotype-matched human mAb, targeting a protein not expressed in the brain, served as control-mAb. In total, we used a set of 4 LGI1 mABs with LRR-mAb02, LRR-mAb03, and EPTP-mAb12 from one patient and EPTP-mAb04 from a second patient. Despite the overall similarity of their binding characteristics, previous studies reported a relatively higher binding efficacy for LRR-mAb02 and EPTP-mAb12.^21^ In this line, while performing the electrophysiologic experiments under all 4 mAbs (separately), we conducted immunocytochemistry and structured illumination microscopy (SIM) experiments using only LRR-mAb02 and EPTP-mAb12. To achieve long-term effects of antibody treatment, we incubated the neuronal cultures 7 days before the experiment and with an additional mAb-containing medium exchange 1 day before the experiment with control-, LRR- (mAb02 and mAb03^21^), or EPTP-mAb (mAb12, mAb04^21^) at a final concentration of 10 µg/mL in fresh culture medium. The analysis of LRR-mAb02 and EPTP-mAb12 data was performed separately from that of LRR-mAb03 and EPTP-mAb04, as they were acquired using distinct batches of cell culture preparations.

### Electrophysiologic Recordings

Whole-cell patch-clamp recordings were obtained from cultured neurons at days in vitro (DIV) 17 to DIV21 and 24–32 hours after the last application of mAb. For this, a coverslip was transferred from culture medium to a submerged recording chamber and perfused with artificial CSF containing (in mM) 138 NaCl, 20 Glucose, 10 HEPES, 2.5 KCl, 1.2 Mg_2_SO_4_, and 2 CaCl_2_, pH 7.4, at room temperature with a perfusion rate of 2–3 mL/min. Electrophysiologic recordings were conducted by a HEKA EPC-10 amplifier with a sampling rate of 20–100 kHz and low-pass filtered at 2.9 kHz using the amplifier's Bessel filters. Excitability of the neurons was measured in current clamp mode with a K-gluconate–based intracellular solution containing (in mM) 150 K-gluconate, 10 HEPES, 10 NaCl, 3 MgATP, 0.3 Na_2_GTP, and 0.05 ethylene glycol-bis(β-aminoethyl ether)-N,N,N',N'-tetraacetic acid, adjusted to pH 7.3 with KOH. Membrane potential values (V_m_) were corrected offline for liquid junction potential (−8 mV). Continuous somatic current injections were recorded at the resting potential of the neurons. Ionotropic glutamate and gamma-aminobutyric acid (GABA) receptors were blocked with 0.5–0.75 mM kynurenic acid and 20 µM bicuculline, respectively. Membrane response and action potential (AP) firing were measured by applying depolarizing current steps from 0 to 250 pA with an increment of 10 pA and a duration of 1 second per step.

### Data Analysis

For input-output curves, the number of APs was plotted against the injected current size of 1-second duration. The rheobase represents the minimal current step required for eliciting an AP. AP latency refers to the time from the beginning of the current step pulse to the peak of the first evoked AP at rheobase current. AP threshold refers to the V_m_ at which an AP is initiated, by considering a minimal AP slope (dV_m_/dt) of 15 V/s. The slope of the depolarizing ramp was calculated by fitting a linear line to the V_m_ trace between 100 and 900 ms at the subthreshold current injection step before rheobase. Input resistance was determined by measuring the steady-state voltage deflections of hyperpolarizing current steps from −100 to −10 pA and calculating the slope of the linear fit.

For each 2 spikes, the instantaneous firing frequency (IFF) was calculated as 1/ISI, where ISI denotes their interspike interval. The initial IFF (*f*_0_) refers to that of the first 2 spikes at each current step; i.e., 1/ISI_1_. The mean IFF was computed by averaging over all 1/ISIs presented during each current step. To estimate the dynamics of IFF over time, i.e., *f*(t), and its steady-state level, i.e., *f*_ss_, we used a standard method,^23^ where the *f*_ss_ was computed by averaging *f*(t) over the last 200 ms of the current step. We quantified the spike frequency adaption (SFA) effect by using 2 different indices^23,24^ as follows: (1) SFA^relative^ = (*f*_0_ − *f*_ss_)/*f*_0_ and (2) SFA^accomodation^ = ISI_last_/ISI_1_, where ISI_last_ refers to the ISI of the last 2 spikes. For both measures, a higher SFA index shows a stronger effect of SFA; i.e., more attenuation of *f*(t) toward the end of the current step, compared with *f*_0_. To compute the effective time of SFA for gaining up to adapt (i.e., attenuate) firing frequency, we computed the time at which the *f*(t) was reduced by 50% relative to *f*_ss_; i.e., the time to reach (*f*_0_ + *f*_ss_)/*2*.

### Immunocytochemistry of Hippocampal Cultures

To investigate K_v_1.1 channel clustering and density at the AIS, hippocampal neuron cultures were incubated live with monoclonal LRR- (mAb02), EPTP- (mAb12), or control-mAb for 7 days at 37°C as described above. Three coverslips were used for each group. After fixation at DIV14 with 4% paraformaldehyde, cells were incubated in blocking buffer (phosphate buffered saline [PBS], 10% normal goat serum, and 0.1% Triton X-100) for 1 hour at room temperature. Primary antibodies (rabbit anti-K_v_1.1, MSFR103610, Nittobo; mouse anti-ankyrinG, N106/36, Merck) were diluted 1:200 in PBS, 1% normal goat serum, and 0.1% Triton X-100 and incubated overnight at 4°C and then thoroughly washed in PBS. CF568 goat anti-mouse and AF640R goat anti-rabbit secondary antibodies at 1:200 dilutions were incubated for 2 hours at room temperature to visualize ankyrinG and K_v_1.1, respectively. Nuclei were stained using 4',6-diamidino-2-phenylindole. The coverslips were then mounted with Fluoromount-G.

A series of superresolution fluorescent images were obtained with a lattice structured illumination microscope (Elyra 7; Carl Zeiss Microscopy GmbH), equipped with a 63× oil immersion objective lens (NA 1.4; Carl Zeiss Microscopy GmbH), a laser system containing 405 nm (50 mW, diode laser), 488 nm (500 mW), 561 nm (500 mW), and 640 nm (500 mW) lasers, and 2 scientific complementary metal oxide semiconductor (sCMOS) cameras (pco edge). Approximately 10 positions per coverslip were randomly selected for imaging, and the experimenter was blinded to the treatment groups. The laser intensities were set to 1% for the 561-nm laser and 2% for the 640-nm laser to obtain a range of fluorescence values high enough for the lattice SIM algorithm to reconstruct the labeled structures. Image stacks of optical sections were acquired with 0.1 µm z-steps, and each z-plane was hereby reconstructed out of 13 phases allowing for 3-dimensional reconstructions. SIM images were rendered as 3D volume structures with Imaris 9.1.2 software (Bitplane). To define the structure and volume of the AIS as the region of interest, first, a Gaussian filter with a surface area detail level of 0.2 µm was applied (smoothing), and then, a mask of the AnkG staining was created by using the automatic background subtraction algorithm of Imaris and setting a threshold for the signal intensity. The AnkG mask was used as a reference for K_v_1.1 colocalization. K_v_1.1 clusters were also detected with the automatic threshold but without smoothing. Based on these 3D reconstruction results, we extracted the number, center position (3D coordinates), and volume of individual K_v_1.1 channel clusters along AIS and the position of visually detected closest cluster to soma.

To analyze the density of K_v_1.1 channel clusters along AIS, we computed both the total number and total volume of K_v_1.1 clusters relative to the AIS volume. To estimate how far each cluster is located from the most proximal site of the AIS (or simply, AIS start) and the density maps, we first projected the 3D cluster positions to x and y coordinates, where the closest detected cluster to soma (Cls1) has an x-position of zero. As some of the AISs had a curved shape, we virtually straightened each AIS by using a robust quadratic curve fitting to the 2D position data (*fit* function of Matlab 2020a) and calculated the distance of each cluster to the AIS start (δ) along the fitted curve using the first derivatives and then *hypot* and *trapz* functions of Matlab. This straightening allowed us to represent the cluster position data across a 2D space along the longitudinal (zero refers to the Cls1 position) vs width dimensions of the AIS. To compute the density maps, we first pooled (i.e., superpositioned) the cluster positions of all straightened AISs in this 2D space and then constructed and plotted the maps using *hist3* (16 vs 11 bins) and *pcolor* functions of Matlab.

To investigate the spatial distribution in more detail, we further broke down the cluster density analysis into 3 AIS sections (of 4 µm length): proximal, middle, and distal. This sectioning was performed based on the distance of cluster positions to Cls1 (see previous paragraph), which were dominantly located within the 12-µm range. For each AIS, for the clusters located in each section (according to their δ), we again computed the number and the total volume of clusters relative to the AIS volume as well as the mean Euclidian pairwise distance (based on the 3D position data) of each cluster to the others within its corresponding section.

### Neuron Model

To investigate the effect of mAbs on neuronal subthreshold and firing response through altering K_v_1 channel properties, we used the simulations of a single-compartment model of the membrane potential of a hippocampal neuron's soma/AIS. Our aim was to provide mechanistic insights into the underlying biophysical mechanisms of our experimental results. The biophysical Hodgkin-Huxley (HH) type neuron model includes the transient Na^+^ current (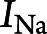) and the delayed rectifier K^+^ current (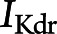) for AP generation, the leak current (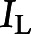), the rapidly activating and inactivating A-type K^+^ current (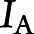), and the Ca^2+^-activated K^+^ current (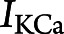) contributing to SFA. To build the model and set its parameters, we mainly followed previous studies.^25-28^ In addition, we added to the model a D-type K+ current (I_D_), mediated by fast activating and slowly inactivating K_v_1 channels; this current has been reported for both hippocampal and cortical neurons.^29-32^ Note that our preliminary analysis revealed that this model with this typical ionic channels provides a sufficiently rich biophysical framework to focus particularly on investigating the key mechanisms underlying our experimental observations. Moreover, as recent studies showed that LGI1 molecules form strong clustering with several K_v_1 channel subunits, but have no direct interaction with AMPARs^33^; therefore, to provide a clearer link between the model behavior and our experimental data recorded from soma, we focused on modeling the soma/AIS without incorporating the AMPARs.

The current balance equation of the membrane potential (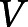) is as follows:
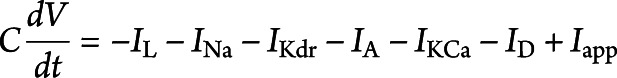
Where 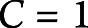, 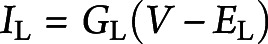 with 
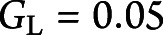
 and 
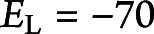
, and 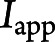 is the applied current. The ionic currents are modeled as: 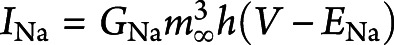, 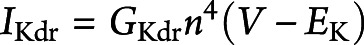, 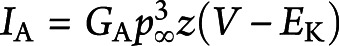, 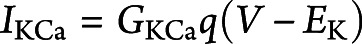, and 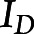, where 
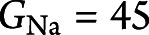
, 
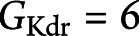
, 
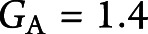
, 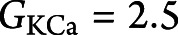, 
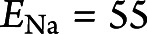
, and 
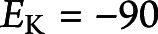
. In consistent with available experimental data,^29,34^ we modeled the D-type K^+^ current as 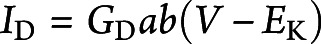, where 
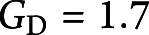
, and 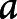 and 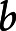 are the activation and inactivation variables of the K_v_1 channel, formulated by HH-type equations. The gating variables (except 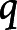) were modeled as 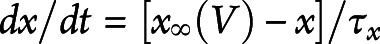 and 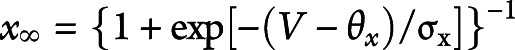, where 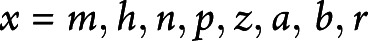 (for 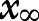, 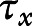, or both), 
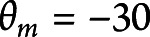
, 
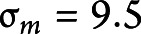
, 
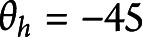
, 
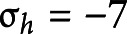
, 
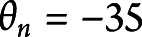
, 
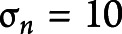
, 
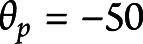
, 
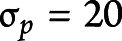
, 
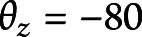
, 
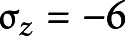
, 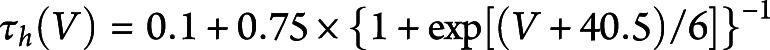, 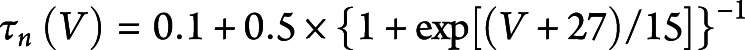, and 
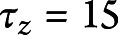
. For 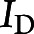, we set 
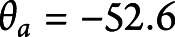
, 
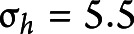
, 
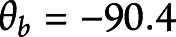
, 
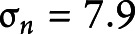
, 
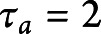
, and 
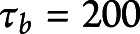
, in consistent with available, corresponding hippocampal data.^29,30^ The activation variable of 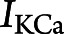 is a function of the somatic Ca^2+^ concentration level, i.e., 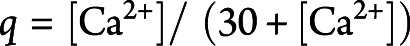, whose kinetics obey 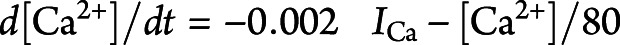
^26,27^, where 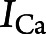 is the inward current mediated by high-voltage-gated calcium channels (i.e., mainly during spikes): 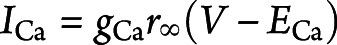, where 
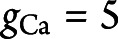
, 
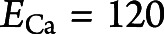
, 
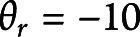
, and 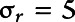.^26,27^ Throughout the article, the units that we used for specific membrane capacitance (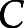), reversal potentials (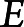), maximal conductance (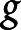), time constants (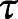), 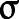, and 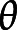 quantities are µF/cm^2^, mV, mS/cm^2^, ms, mV, and mV, respectively.

Before stimulating the neuron model, we first simulated it with 
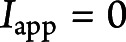
 µA/cm^2^ for a sufficiently long time (20 seconds) to ensure that all variables reach their steady-state values at the rest potential. This was followed by injecting a positive current step of 
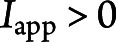
 µA/cm^2^ to the soma to investigate the neuron response under different conditions (see Results).

### Statistical Analysis

Statistical analyses were performed using OriginPro 2019 and Matlab 2020a. All data are reported as mean ± SEM, if not stated otherwise. The Shapiro-Wilk test was used to test for normality. The F-test was used to test for homogeneity of variances. For the Shapiro-Wilk test and F test, a significance level of 0.05 was used. For multigroup comparisons, one-way analysis of variance (ANOVA) was used for normally distributed data and the Kruskal-Wallis test for non-normally distributed data. Following a significant result in the ANOVA, post hoc pairwise comparisons were performed using the Fisher's Least Significant Difference (LSD) test. Following a significant result in the Kruskal-Wallis test, post hoc pairwise comparisons were performed using the Mann-Whitney *U* test (MWU). Curve permutation tests were performed by comparing the difference in the area under the curve of the group-averaged curves, with those obtained after shuffling the individual curves across groups, with 1 million times repetition; see Ref. 35 for more details. To statistically compare the cluster density and distance parameters between groups at proximal, middle, and distal sections, we adapted the permutation test of Cohen, thereby accounting for multiple comparison problem. Details of the applied statistical tests, *p* values, and sample sizes are provided in figure legends.

### Standard Protocol Approvals, Registrations, and Patient Consents

Experiments using animals were performed in accordance with the ARRIVE guidelines for reporting animal research,^22^ and the experimental protocol was in accordance with European regulations (Directive 2010/63/EU) and was approved by the local ethics committee at the University of Jena.

### Data Availability

Data not provided in the article because of space limitations may be shared (anonymized) at the request of any qualified investigator for purposes of replicating procedures and results.

## Results

### LRR- but Not EPTP-Specific Monoclonal Antibodies Increase the Neuronal Firing Rate

**Figure 1:**
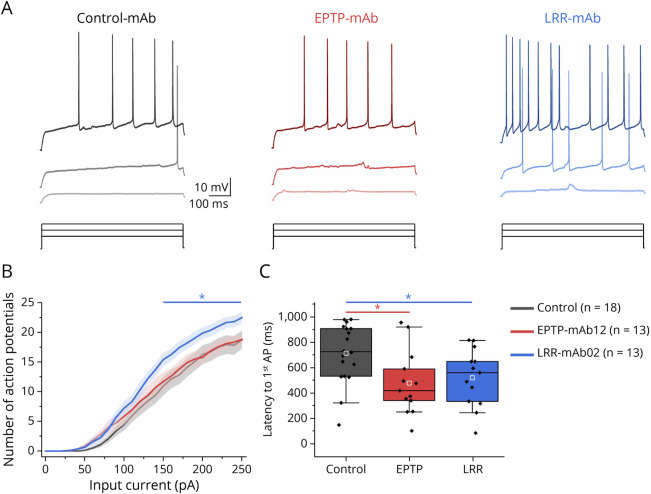
LRR- But Not EPTP-mAb Increases the Neuronal Firing Rate in Primary Hippocampal Cell Cultures (A) Membrane potential traces of cell responses to 1-second depolarizing current steps (bottom: +40 pA, middle: +90 pA, and top: +130 pA) from neurons treated with control-mAb (n = 18 cells, left panel, black), EPTP-mAb12 (n = 13, middle panel, red), or LRR-mAb02 (n = 13, right panel, blue) for 7 days. (B) Neurons fire more action potentials (APs) with increasing step currents from 0 to 250 pA under LRR-mAb treatment (whole-curve permutation test: P [EPTP vs control] = 0.313; P [LRR vs control] = 0.023 [blue *]). (C) The LRR and EPTP mAb treatment cause a reduction in the latency to 1st AP at the individual rheobase current (one-way ANOVA followed by the Fisher LSD post hoc test, P [EPTP vs control] = 0.010 [blue *]; P [LRR vs control] = 0.033 [red *]). Box plots show the median, 25th, and 75th percentiles; whiskers indicate the 10th and 90th percentiles; open squares represent the mean, and each filled dot represents single cells. EPTP = epitempin; LRR = leucine-rich repeat.

We treated primary hippocampal neurons with mAbs against either the LRR (mAb02) or EPTP (mAb12) domain or with nonreactive isotype control mAb for 7 days and then performed whole-cell patch-clamp recordings in current clamp mode with incremental injection of 10 pA current steps to the soma for 1 second. Of interest, when compared with the control mAb, an increase in the number of APs was observed in neurons preincubated with the LRR-mAb02 but not with the EPTP-mAb12 (Figure 1, A and B). However, the latency of the first AP occurrence at the rheobase current step showed earlier spiking for both LRR-mAb02– and EPTP-mAb12–treated neurons (Figure 1C, control: 712.2 ± 55.5 ms, EPTP: 478.2 ± 70.1 ms, LRR: 520.3 ± 63.0 ms; one-way ANOVA). These changes of neuronal excitability reflect mAb-induced alterations because we did not find a significant change in the intrinsic membrane properties of the recorded neurons (eFigure 1, links.lww.com/NXI/A819). When repeating the experiments with another set of autoantibodies against LGI1 domains, namely EPTP-mAb04 and LRR-mAb03 with less binding efficacy,^21^ we obtained qualitatively similar results (eFigure 2A-C, links.lww.com/NXI/A819). In sum, these results indicate an increased firing activity of LRR-mAb–treated neurons, without a marked change in their intrinsic membrane properties.

### LRR-Specific Antibodies Enhance Initial Firing and Spike-Frequency Adaptation

**Figure 2:**
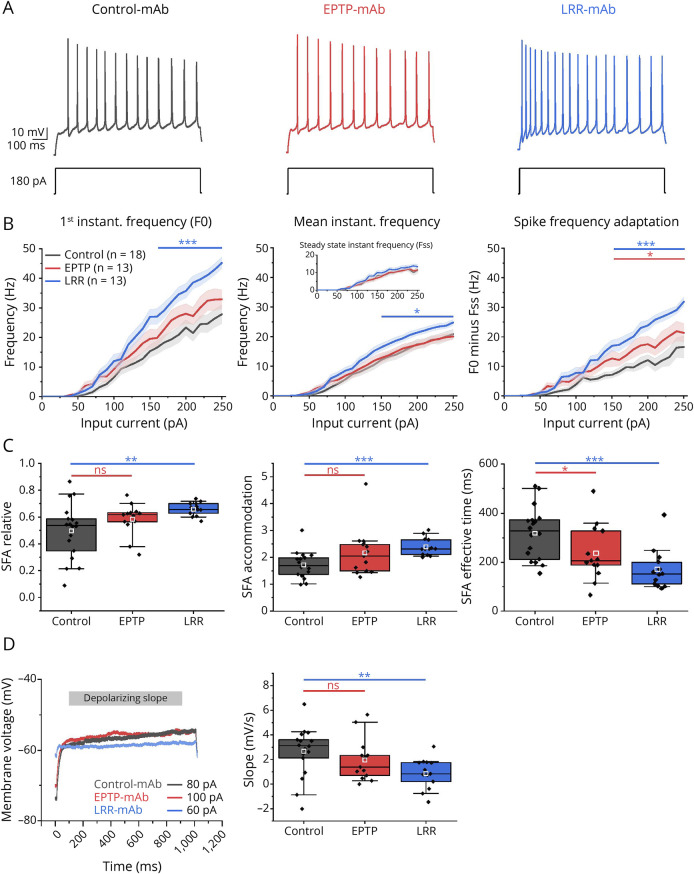
LRR-mAb Enhances Initial Firing and Spike-Frequency Adaptation Effect (A) Sample traces of spiking behavior of neurons treated with control- (left), EPTP-(middle), or LRR-mAb (right) in response to a step current of +180 pA. (B) LRR-mAbs induce a more pronounced enhancement of, in particular initial, firing frequency (*f*) of neurons, particularly at current steps above 150 pA. (B, left) Initial instantaneous frequency, *f*_0_ (whole-curve permutation test: P [EPTP vs control] = 0.087, P [LRR vs control] = 0. [blue ***]). (B, middle) Mean instantaneous frequency (P [EPTP vs control] = 0.338; P [LRR vs control] = 0.021 [blue *]). Inset shows the levels of the instantaneous frequency at steady state (*f*_ss_), which remained unaffected (P [EPTP vs control] = 0.485; P [LRR vs control] = 0.137). (B, right) Absolute spike frequency adaptation measured as *f*_0_ − *f*_ss_ shows a potent increase under LRR-mAb (whole-curve permutation test: P [EPTP vs control] = 0.041 [red *]; P [LRR vs control] = 0.0001 [blue ***]). (C) Neuronal firing frequency undergoes a stronger and faster adaptation (attenuation) after LRR-mAb treatment. Spike frequency adaptation was quantified by SFA^relative^ (C, left) and SFA^accomodation^ (C, middle), where a higher index represents a stronger attenuation of instantaneous frequency over time, per current step. (C, left) SFA^relative^ (one-way ANOVA followed by the Fisher LSD post hoc test: P [EPTP vs control] = 0.117; P [LRR vs control] = 0.005 [blue **]). (C, middle) SFA^accomodation^ (Kruskal-Wallis test followed by the MWU post hoc test: P [EPTP vs control] = 0.143; P [LRR vs control] = 0.0002 [blue ***]). (C, right) SFA effective time (P [EPTP vs control] = 0.043 [red *]; P [LRR vs control] = 0.0007 [blue ***]). (D) LRR-mAbs cause a remarkable drop in the I_D_ current-dependent slope of the ramp-like depolarization in the subthreshold response. (D, left) Sample voltage traces showing the subthreshold response at the corresponding prerheobase current step for each group. (D, right) The slope of ramp for all individual neurons (one-way ANOVA followed by the Fisher LSD post hoc test: P [EPTP vs control] = 0.271; P [LRR vs control] = 0.007 [blue **]). Box plots show the median, 25th, and 75th percentiles; whiskers indicate the 10th and 90th percentiles; open squares represent the mean, and each filled dot represents a single cell value. EPTP = epitempin; LRR = leucine-rich repeat; MWU = Mann-Whitney U test; ; ns = non-significant; SFA = spike frequency adaption.

K_v_1 channels mediate a slowly inactivating K^+^ current (I_D_), which can effectively regulate the early IFF of both hippocampal and cortical neurons.^29-32^ Because (1) LGI1 binding triggers a protein complex including K_v_1 channels and (2) LGI1 molecules can selectively prevent K_v_1 inactivation mediated by the auxiliary protein Kvβ,^36^ we hypothesized that mAbs that interfere with the LGI1-K_v_1 complex may increase the IFF of treated neurons. To test this hypothesis, we first computed the initial IFF (*f*_0_) as 1/ISI_1_, where ISI_1_ is the interspike interval between the first 2 spikes at each step current. Consistent with the increased excitability (Figure 1), we found an increase in *f*_0_ of LRR-mAb02 and, to a lesser extent, of EPTP-mAb12–treated neurons (Figure 2, A and B, left). We obtained similar results of LRR-mAb02 induced effects when using the mean IFF (i.e., by averaging over all 1/ISIs; Figure 2B, middle), whereas the steady-state level of IFF (*f*_ss_) remained unaffected between all experimental groups (inset in Figure 2B, middle); *f*_ss_ was computed based on the spikes within last 200 ms.

These results motivated us to quantify the effect of SFA mechanism, which enforces a progressive reduction of the frequency of AP discharges over time. In particular, we hypothesized a pronounced increase in the firing adaptation in neurons treated with LRR-mAb02 because of their elevated *f*_0_. To investigate this point, we subtracted *f*_ss_ from *f*_0_ for each step current: this parameter showed a pronounced increase in neurons incubated with the LRR-mAb02, which was again more pronounced compared with effects induced by the EPTP-mAb12 (Figure 2B, right). This indicated a stronger adaptation of these cells' firing frequency, manifested also in their higher SFA indices, which we quantified with 2 different measures as (f_0_ − f_ss_)/f_0_ (Figure 2C, left; SFA^relative^) and ISI_last_/ISI_1_ (Figure 2C, middle; SFA^accomodation^). In addition, the effective time of the SFA to adapt ISIs (i.e., the time required for IFF to drop from *f*_0_ to (*f*_0_ + *f*_ss_)/*2*) was potently reduced (Figure 2C, right, control: 316.9 ± 25.0, EPTP: 236.6 ± 31.6, and LRR: 171.5 ± 22.7), pointing to a faster decrease in ISIs under LRR-mAb.

A characteristic of D-type K^+^ channels such as K_v_1.1 is fast activation in response to cell depolarization from resting potential but a slow inactivation within seconds.^30^ In this line, during a step current, the I_D_ current activation dampens the subthreshold depolarization at the stimulus onset, which then undergoes a slow progressive build-up (ramp-like depolarization) due to slow inactivation of K_v_1 channels. Accordingly, as a measure of K_v_1 channel strength, we analyzed the slope of this ramp-like subthreshold response at the prerheobase subthreshold current step, similarly to previous studies.^20,29,30^ The slope was strongly reduced in neurons treated with LRR-mAb02 but not EPTP-mAb12 (Figure 2D), reflecting K_v_1 channel dysfunction.

We observed qualitatively similar domain-specific effects for the second set of antibodies (LRR-mAb03 and EPTP-mAb04), including an increase in *f*_0_, a slightly increased SFA effect, and a reduction in the depolarizing slope only for LRR-mAb03 (eFigure 2, D–F, links.lww.com/NXI/A819). Of note, these effects were not as pronounced as the first set, which may reflect the relatively lower binding efficacy of LRR-mAb03 and EPTP-mAb04.^21^ In sum, these results indicate that LRR- more than EPTP-specific mAbs enhance the SFA effect, by impairing the neuron's control on its early-phase of firing frequency.

### A Biophysical Neuron Model Accounts for Major Experimental Observations

**Figure 3:**
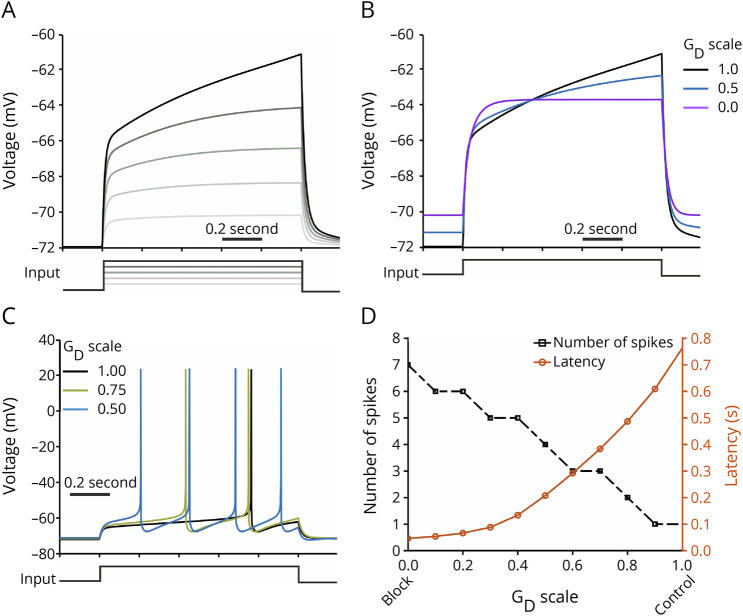
Biophysical Neuron Model Identifies Disrupted D-Type K^+^ Current (I_D_) Accounting for the Observed Higher Neuronal Excitability (A) Presence of a slow ramp-like depolarization in the simulated, subthreshold somatic response to the current step (1 second), due to activation of I_D_ current. Current steps: 0.1, 0.2, …,∼0.5. (B) The slope of ramp-like depolarization reduces by lowering G_D_ density. The membrane potential traces were shown at the prerheobase current step at each simulated G_D_ scale; 1 × G_D_: control (I_app_ = 0.48), 0.5 × G_D_: half-blockage of K_v_1 channels (I_app_ = 0.35), 0 × G_D_: complete blockage (I_app_ = 0.23). (C and D) Neuron model fires more spikes and exhibit shorter first spike latency at smaller G_D_. (C) Example voltage traces of 3 different G_D_ scales. I_app_ = 0.53. (D) The number of evoked spikes and the latency to first spike for a complete range of G_D_ scale.

Hitherto, our data pointed to a pronounced increase in *f*_0_ but not *f*_ss_ leading to a stronger SFA effect predominantly in LRR-mAb–treated neurons. Next, we aimed at mechanistically investigating whether an isolated reduction in the K_v_1 channel conductance in the neuron model can explain our experimental findings with mAb against LRR, including (1) a decreased AP latency (Figure 1C), (2) an increased SFA index (Figure 2C), and (3) a more shallow voltage slope during the prerheobase current step injection (Figure 2D; see also eFigure 2F, links.lww.com/NXI/A819). To do this, we investigated a single-compartment model of a hippocampal neuron with HH-type channel models,^25,26^ including a fast activating but slowly inactivating K^+^ current emulating K_v_1-mediated I_D_ (see Methods).^29,30,32^ We modeled the effect of LGI1 mAb as a reduction in the maximal conductance of the K_v_1 channel (G_D_). In analogy to our experiments, we stimulated the model by injecting current steps for 1 second.

**Figure 4:**
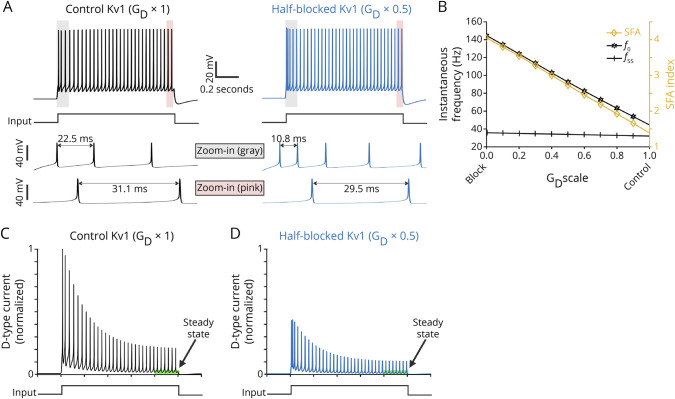
Biophysical Neuron Model Identifies Disrupted D-Type K^+^ Current (I_D_) Accounting for the Observed Higher Initial Frequency and SFA Effect (A and B) Lowering G_D_ leads to a remarkable increase in the initial instantaneous frequency (*f*_0_) and SFA^accomodation^ index with a minor effect on the steady-state value *f*_ss_. The representative results were simulated at I_app_ = 2.5. (A) Example voltage traces for conditions of G_D_ scale = 1 (control) and 0.5. (B) The SFA^accomodation^ index, *f*_0_, and *f*_ss_ values for a complete range of G_D_ scale. (C) The time course of I_D_ current during the current step, related to the voltage traces in (A). (C and D) Lowering G_D_ decreases D-type K^+^ current (I_D_), thereby impairing the ability of the neuron model to constrain its initial firing frequency (compare corresponding panels in [A]). Current traces were normalized to the maximum value across the 2 conditions. SFA = spike frequency adaption.

The slow ramp-like depolarization in the subthreshold response of the simulated control neuron exhibited a steeper slope at higher current step sizes (Figure 3A). The slope of this ramp, quantified at the prerheobase current step, showed a strong dependency on G_D_: ∼2 mV/s under control (black), and ∼1 and 0 mV/s for G_D_ scales of 0.5 (half-blockage of K_v_1-channel; blue) and 0 (complete blockage; magenta), respectively (Figure 3B; see also Figure 2D). The resting potential increased slightly by reducing G_D_, consistent with previous reports^30^ and our data (e.g., eFigure 1C, links.lww.com/NXI/A819). Moreover, we observed that reducing G_D_ shortens effectively the latency of first AP at the rheobase current step and causes an increase in the number of APs (Figure 3, C and D), consistent with our experimental findings (Figure 1, B and C). Furthermore, the neuron model's *f*_0_ but not *f*_ss_ starkly increased after reducing G_D_ (Figure 4A), as observed experimentally with LRR mAb (Figure 2, B and D). In this line, the SFA index of the neuron model underwent a potent increase at smaller G_D_ scales (Figure 4B), corroborating our experimental data with LRR mAb (Figure 2C). The larger I_D_ amplitudes during the initial phase of firing (Figure 4, C and D) provide an explanation for the stronger effect of I_D_ reduction on the frequency during the initial phase of firing and thus for the increased SFA index observed with LRR mAb (Figure 2, B and C). These modeling results demonstrate that an isolated reduction in the conductance of K_v_1 channels can explain our findings after LRR-mAb treatment.

### LRR-Specific Antibodies Cause an Abnormal Redistribution of K_v_1.1 Clusters at the Distal Axon Initial Segment

**Figure 5:**
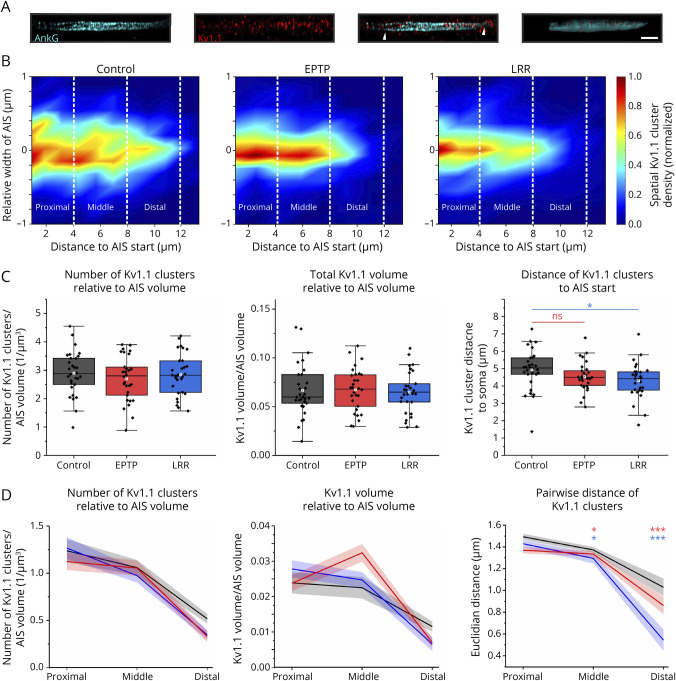
LRR-Specific Antibodies Cause an Abnormal Redistribution of K_v_1.1 Clusters at the Distal AIS (A) Sample images of ankyrinG staining, demonstrating the AIS, K_v_1.1 channel clusters and the overlay of both detections. Arrowheads indicate the beginning and end of the AIS used for 3D reconstruction. Right*:* 3D reconstruction of AIS and colocalized K_v_1.1 clusters after SIM imaging. Scale bar = 2 µm. (B) The overall density maps of K_v_1.1 clusters over AIS, showing a marked shrinkage of their spatial dispersion across AIS, after LRR-mAb treatment (control: n = 30, EPTP-mAb: n = 30, and LRR-mAb: n = 31 AIS). The map of each group was normalized to its maximum value. (C) The number (left, one-way ANOVA followed by the Fisher LSD post hoc test, P [EPTP vs control] = 0.313; P [LRR vs control] = 0.796) and total volume (middle, one-way ANOVA followed by the Fisher LSD post hoc test, P [EPTP vs control] = 0.914; P [LRR vs control] = 0.747) of K_v_1 clusters remained unchanged. LGI1 mAbs redistribute K_v_1 clusters towards the proximal site of the AIS (right). (D) LRR-mAb affects K_v_1.1 cluster density largely at the distal AIS. The analysis was performed per each of the proximal, middle, and distal sections of the AIS (as depicted in B). Left and middle: the number and total volume of K_v_1 clusters relative to AIS volume. Right: the mean Euclidian distance of each cluster to the others within each AIS section. Data presented as median ± SEM. The tests were performed based on the permutation test of Cohen, thereby accounting for multiple comparison problem (*p < 0.05; ***p < 0.001). AIS = axonal initial segment; ANOVA = analysis of variance; EPTP = epitempin; LGI1 = leucine-rich glioma inactivated protein 1; LRR = leucine-rich repeat; NS = non-significant; SEM = standard error of the mean.

**Figure 6:**
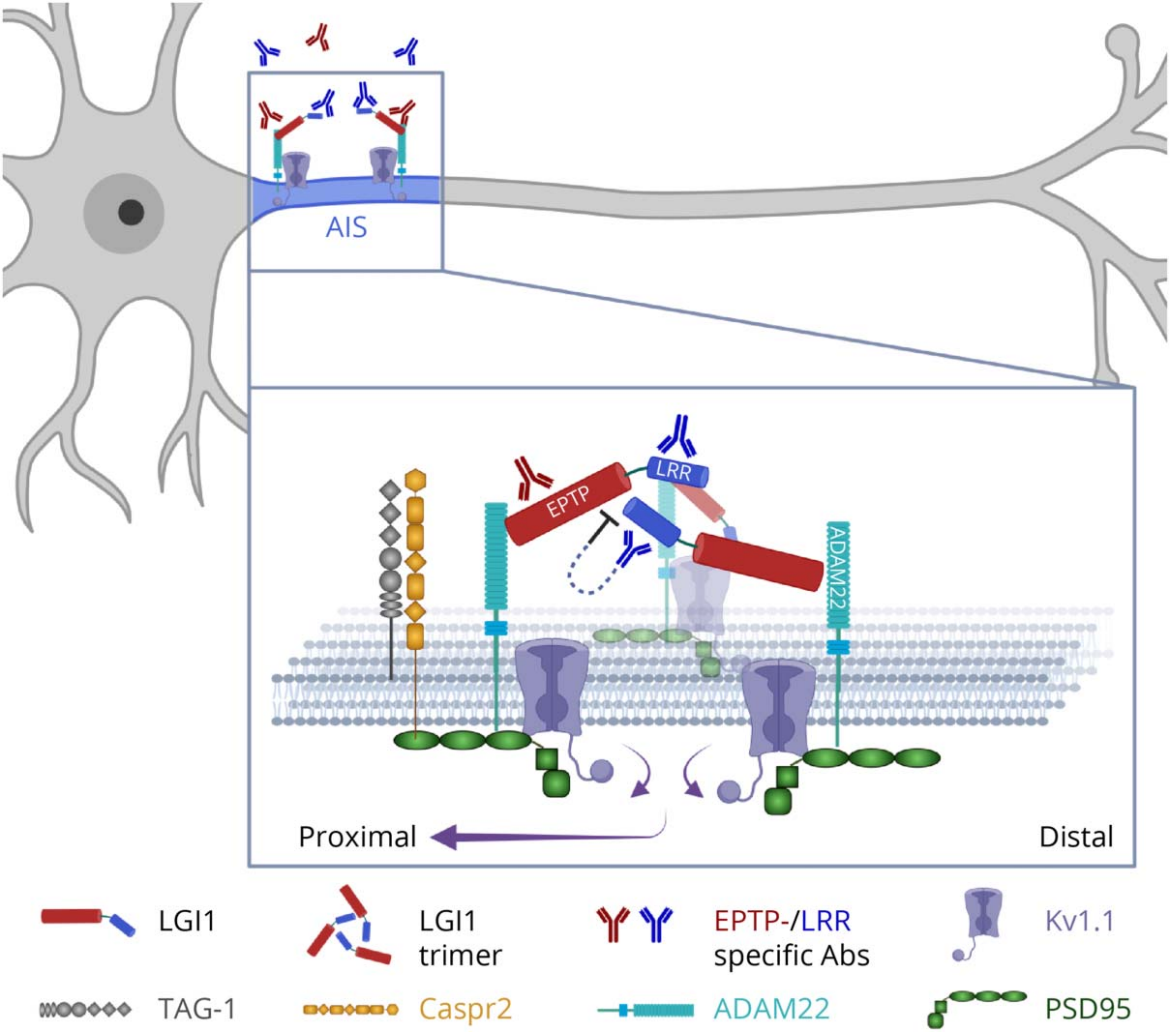
LRR-mAb Disturb Higher-Assembly LGI1-ADAM22-Kv1 Complexes at the AIS and Trigger a Proximal Shift of K_v_1.1 Channels The scheme depicts the model of a 3:3 heterohexamer where LGI1 binds ADAM22 in a *cis*-conformation, as well as the cell adhesion molecules (e.g., TAG-1 and Caspr2), which anchor these complexes at the AIS membrane, thereby leading to tight clusters of K_v_1.1 channels along the AIS (based on Ref.^13^). LGI1 autoantibodies, in particular LRR-mAb, disrupt the interaction between LGI1 molecules, which may destabilize the tripartite complexes and anchoring of K_v_1.1. This altered cluster organization results in a noticeable redistribution of K_v_1.1 channels toward the proximal site of the AIS and subsequent disturbances in the neuronal control on AP initiation and subthreshold integration. Created with BioRender.com. AIS = axonal initial segment; LGI1 = leucine-rich glioma inactivated protein 1.

Having found a potential role of reduction in K_v_1 channel conductance to explain our results after LGI1 mAb treatments, we then asked whether these autoantibodies affect the K_v_1 channels clustering at the AIS. This is because (1) LGI1 was shown to be enriched at the AIS and colocalized with ADAM22 and K_v_1 channels where they likely form higher-order clusters and regulate the density of K_v_1 channels,^18,37^ (2) epilepsy-associated sequence variations of LGI1 led to impaired trafficking and anchoring at the AIS,^19^ and (3) our experimental and modeling results suggested an impaired K_v_1-channel activity in LRR-mAb–treated neurons (Figures 2–4). Using immunocytochemistry and structured illumination microscopy (lattice SIM) after a long-term incubation of hippocampal neurons with LRR-mAb02 or EPTP-mAb12, we performed a 3D reconstruction of K_v_1.1 channel clusters in ∼30 AISs for each group (Figure 5A). Strikingly, the LRR-mAb02 and, to less extent, EPTP-mAb12 led to a spatial redistribution of the cluster density toward the most proximal site of the AIS (simply, AIS start) and less dispersion over AIS (Figure 5, B and C, right), despite the unchanged number and total volume of K_v_1.1 clusters relative to AIS volume (Figure 5C, left and middle). This effect appeared to be stronger, in particular, toward the end of AIS (Figure 5B). This is important because the AP is effectively triggered at the distal AIS.^38,39^ Of note, the AIS volume did not change per se between groups (control: 13.04 ± 0.69, EPTP: 12.85 ± 0.80, LRR: 12.38 ± 0.62 µm^3^; *p* = 0.792, one-way ANOVA). To investigate this observation in more detail, we broke down the cluster density analysis into three AIS sections (of 4 µm) relative to the soma's position; proximal, middle, and distal (Figure 5, B and D). Consistent with density maps (Figure 5B), the number and volume of clusters tended to be lower at the distal AIS under both LGI1-mAbs (Figure 5D). Specifically, computing the mean pairwise distance between clusters within each section revealed a noticeable reduction at nonproximal sections, in particular at the distal AIS, which was more pronounced under LRR-mAb02 than EPTP-mAb12 (Figure 5D, right). This closer distance of clusters reflects a shrinkage of their spatial dispersion over AIS (see also Figure 5B). Taken together, these results indicate a marked abnormal redistribution of K_v_1 channels under LRR-mAb (Figure 6), in particular, at the distal AIS. Considering the importance of K_v_1 channels at the AIS for cellular excitability,^40,41^ our data suggest that the observed K_v_1 channel redistribution contributes to the observed enhanced firing and SFA effect.

## Discussion

We here describe epitope-specific effects of antibodies against LGI1 on intrinsic neuronal excitability. We found that LRR-directed mABs can directly affect neuronal excitability, manifested as an increase in the initial frequency, SFA strength, and number of evoked APs. The effects of EPTP-specific mABs were less pronounced and mainly resulted in decreased firing latency of the first AP. The combination of our experimental data and a biophysical neuron model highlighted reduced K_v_1-channel mediated D-type K^+^ current (I_D_) as an explanation for this increased excitability. This, in turn, causes a stronger and more rapid SFA, even when the density of channels mediating the adaptation, such as Ca^2+^-activated or M-type K^+^ channels,^25,26,30^ remained unchanged in the model. Furthermore, the K_v_1.1 channel density at the distal AIS underwent a noticeable redistribution under LRR-mAb and, to a lesser extent, under EPTP-mAb, suggesting a reduced neuron's control on the AP initiation and subthreshold integration.

These results obtained in primary neurons corroborate observations obtained in rat hippocampal slice cultures using similar patient-derived mAbs against LRR and EPTP, which have been attributed to reduced K_v_1 channel–mediated I_D_ current.^42^ This has been substantiated here and in slice cultures^20^ by reduced slope of ramp-like depolarization of subthreshold response at prerheobase current step injection (see also Refs. 20,29,30 for using this measure) and in recent studies by diminished effects of the K_v_1.1-specific channel blocker dendrotoxin K.^20,42^ This view is also supported by LRR-specific antibodies that may prevent the interaction of LGI1 to the K_v_β subunits, resulting in the transformation of slowly inactivating D-type currents into rapidly inactivating A-type currents, as shown in an in vitro model.^36^ This hypothesis would support our observations in the significantly reduced K_v_1.1-dependent depolarization ramp and the underlying conductance G_D_, as shown in the computational model (Figure 2D, Figure 3, A and B). A direct link of the LGI1 LRR domain to the intracellularly located K_v_β, however, has not yet been elucidated and requires further clarification.

Recent work showed that LRR-specific mAbs lead to LGI1-ADAM22/23 internalization^21^ and suggested that they may affect clustering of K_v_1 channel complexes at the AIS and disrupt AP initiation and shape. In this line, LGI1 knockout resulted in subsequent reduction of K_v_1.1 expression at the AIS by about 50% and induced neuronal hyperexcitability with increased AP firing.^18,19^ Using passive transfer of patient-derived polyclonal LGI1 antibodies into mouse brains, we previously showed a reduction of K_v_1.1 at hippocampal cell surfaces and synapses by ∼20%.^16^ However, here, despite a remarkable redistribution of K_v_1.1 channel complexes along AIS, their overall expression at the AIS was unaffected. This discrepancy may be due to the different organization of K_v_1.1-LGI1-ADAM22 clusters at the AIS compared with synapses. This is because, in synaptic clefts, 2:2 LGI1-ADAM22 complexes can build trans-synaptic nanocolumns,^13,37^ including presynaptic K_v_1 channels and postsynaptic density protein-95 (PSD-95) and other membrane-associated guanylate kinases (MAGUK).^43^ At the AIS, recent studies suggested the presence of a 3:3 heterohexamer where LGI1 molecules bridge ADAM22 molecules in a *cis*-conformation on the same membrane.^13,37^ In this line, it has been conjectured that cell adhesion molecules, e.g., transient axonal glycoprotein-1 (TAG-1) and contactin-associated protein-like 2 (Caspr2) molecules, anchor these tripartite LGI1-ADAM22 complexes at the AIS and lead to clustering of K_v_1 channels (Figure 6).^13^ In addition, recent studies demonstrated that LRR mAbs induce internalization of LGI1-ADAM22 complexes, whereas EPTP mAbs inhibited the binding of LGI1 to its receptors ADAM22/23.^21^ Therefore, this discrepancy may also reflect that the autoantibodies affect the cluster organizations at AIS differently than at synapses. Nonetheless, dissecting these hypotheses requires future investigations.

Our imaging data revealed a noticeable redistribution of K_v_1 channels toward the proximal site of the AIS. This effect was more potent under LRR-mAb than EPTP-mAb, suggesting a stronger destabilization of higher-assembly LGI1-ADAM22-K_v_1 complexes after LRR-mAb treatment (Figure 6). APs are effectively triggered at the end of the distal AIS.^38,39,44^ Hence, our observed shrinkage of spatial distribution of K_v_1 clusters and their relatively lesser localization at the distal AIS, compared with control, imply the disturbance of neuron's control on AP initiation and subthreshold integration. In other words, this abnormal distribution of K_v_1 channels may impair the proper clamping of the voltage signal at the distal AIS, which can in turn contribute to the observed hyperexcitability.

It was shown that EPTP-specific mAbs have limited binding affinity in live cell-based assays and acute brain slices,^20,21^ although the injection in mouse brains showed pathogenic effects, although less severe than LRR-mAbs.^21^ This may be due to the use of (1) an artificial experimental model of cultured neurons or brain slice cultures and the direct incubation with the mAbs^20,21^ and/or (2) a relatively short incubation time (∼30–60 minutes) with mAbs before fixation and secondary antibody incubation.^21^ This is because the binding to ADAMs can be largely reduced because of the masked epitope of ADAM22/23-bound LGI1. Indeed, the binding of LGI1 to ADAMs is inhibited if soluble LGI1 is preincubated with EPTP-mAbs.^21^ To relax this limited binding issue, here, we exposed neurons to mAbs for 7 days, which should be a sufficiently long time to allow the cells to produce and secrete LGI1, which is not yet bound to ADAM22 and therefore can be targeted by EPTP-specific mAbs. Moreover, continuous passive transfer of mAbs would be in favor for future investigations to mimic the chronic antibody production of patients.

Previous studies showed simultaneous occurrence of both epitope-specific LGI1 antibodies in patients with LGI1 encephalitis with an increased ratio of LRR-specific IgG.^20^ From our data and other reports investigating epitope-specific effects of LGI1 mAbs,^15,21,42^ LRR-specific IgG might be more effective in inducing hyperexcitability and possibly leading to seizure initiation. However, larger patient cohorts need to be investigated to evaluate the relative contribution of epitope-specific IgG antibodies for disease symptom development.

In our study, the divergent results between LRR- and EPTP-specific mAbs on neuronal excitability were obtained robustly using each of the 2 clones used for these mAbs. Our findings are corroborated by other recent or parallel studies investigating different LRR and EPTP-specific mAbs that also describe a more pronounced effect of LRR-in comparison to EPTP-specific mAbs leading to increased neuronal excitability.^20,42^ In addition to understand antibody-induced pathology at the AIS, it will be another important goal for further studies to address the role of epitope-specific antibodies in the dysfunction and disassembly of the macromolecular complex of LGI1, ADAM22/23, K_v_1 channels, and AMPA receptors at the synapse.^16,20^

## Glossary

AIS: axonal initial segment
AMPA: α-amino-3-hydroxy-5-methyl-4-isoxazolepropionic acid
ANOVA: analysis of variance
AP: action potential
DIV: days in vitro
EPTP: epitempin
HH: Hodgkin-Huxley
IFF: instantaneous firing frequency
ISI: interspike interval
LGI1: leucine-rich glioma inactivated protein 1
LRR: leucine-rich repeat
mAb: monoclonal antibody
MWU: Mann-Whitney *U* test
PBS: phosphate buffered saline
SFA: spike frequency adaption
SIM: structured illumination microscopy
